# Supplementary material for: Clinical evolution of ST11 carbapenem resistant and hypervirulent Klebsiella pneumoniae
Source: Commun Biol. 2021 Jun 1;4:650. doi: 10.1038/s42003-021-02148-4 (PMC8169677; doi:10.1038/s42003-021-02148-4)
Supplement: Supplementary file 2 — Supplementary Information [file 42003_2021_2148_MOESM2_ESM.pdf]

Supplementary Materials

Clinical evolution of ST11 carbapenem resistant and hypervirulent *Klebsiella pneumoniae*

Miaomiao Xie[1#], Xuemei Yang[1#], Qi Xu[1], Lianwei Ye[1], Kaichao Chen[1], Zhiwei Zheng[1], Ning Dong[1], Qiaoling Sun[2], Lingbin Shu[2], Danxia Gu[3], Edward Wai-Chi Chan[1,4], Rong Zhang[2*], Sheng Chen[1*]

Running title: Conjugative MDR and hypervirulent plasmid

[1]Department of Infectious Diseases and Public Health, Jockey Club College of Veterinary Medicine and Life Sciences, City University of Hong Kong, Kowloon, Hong Kong.

[2] Department of Clinical Laboratory, Second Affiliated Hospital of Zhejiang University, School of Medicine, Zhejiang, Hangzhou, China.

[3] Department of Clinical Laboratory, Zhejiang Provincial People's Hospital, Hangzhou, China.

[4] State Key Lab of Chemical Biology and Drug Discovery, Department of Applied Biology and Chemical Technology, The Hong Kong Polytechnic University, Hung Hom, Kowloon, Hong Kong.

# These authors contributed equally.

Corresponding author: Sheng Chen, Email: shechen@cityu.edu.hk from City University of Hong Kong, Kowloon, Hong Kong; Rong Zhang, Email: zhang-rong@zju.edu.cn, Second Affiliated Hospital of Zhejiang University, School of Medicine, Zhejiang, Hangzhou, China.

Supplementary Table S1. Bacterial strains carrying *bla*$_{KPC-2}$-bearing plasmid p17ZR-91-KPC obtained from two hospitals in China and the GenBank.

bla$_{KPC}$-bearing plasmid	Virulence plasmid	Chromosome	Species	ST	K_locus	Country	Date	Source
MK262711.1	-	CP034249.1	*Klebsiella pneumoniae*	ST11	KL112	China	2018	urine
KC757417.2	-	NA	*Klebsiella pneumoniae*	NA	NA	France	2010	NA
MK158080.1	-	NA	*Klebsiella pneumoniae*	NA	NA	China	NA	urine
AP018584.1	-	GCA_003115215.1	*Klebsiella pneumoniae*	ST15	KL62	Viet Nam	2016	NA
AP018830.1	-	NA	*Enterobacter hormaechei*	NA	NA	Myanmar	2015	NA
CP014005.1	-	CP014004.1	*Klebsiella pneumoniae*	ST14	KL2	China	2014	urine
CP017386.1	-	CP017385.1	*Klebsiella pneumoniae*	ST15	KL64	Taiwan	2007	urine
CP021959.1	-	CP021960.1	*Klebsiella pneumoniae*	ST37	KL105	NA	NA	NA
CP024876.1	-	CP024874.1	*Klebsiella pneumoniae*	ST15	KL19	Thailand	2016	sputum
CP024917.1	-	CP024916.1	*Klebsiella pneumoniae*	ST147	KL10	Thailand	2016	rectal swab
CP025966.2	-	CP025963.2	*Klebsiella pneumoniae*	ST273	KL15	China	2017	NA
CP026588.1	+ (CP026587.1)	CP026586.1	*Klebsiella pneumoniae*	ST86	KL2	China	2016	NA
CP028389.2	+ (CP028390.1)	CP028391.1	*Klebsiella pneumoniae*	ST36	KL62	China	2015	NA
CP028553.1	-	CP028555.1	*Klebsiella variicola*	ST1142	KL105	China	2016	NA
CP028717.1	-	CP028716.1	*Klebsiella pneumoniae*	ST15	KL28	China	2017	Sputum
CP034085.1 (pR210-2-CTX)	+ (CP034083.1)	CP034082.1	*Klebsiella pneumoniae*	ST23	KL1	China	2015	NA
HF545434.1	-	NA	*Klebsiella pneumoniae*	NA	NA	Viet Nam	2006	nasal swab
KP987218.1	-	NA	*Klebsiella pneumoniae*	NA	NA	China	2010	cerebrospinal fluid specimen
LT968762.1	-	NA	*Klebsiella pneumoniae*	NA	NA	China	2009	specimen
MG878868.1	-	NA	*Klebsiella pneumoniae*	NA	NA	Taiwan	2013	wound
CP035124.1	-	CP035123.1	*Escherichia coli*	-	-	China	2017	urine
CP018340.1	-	CP018337.1	*Klebsiella pneumoniae*	ST23	KL57	Germany	2014	wound
CP022442.1	-	CP022444.1	*Klebsiella quasipneumoniae*	ST421	KL123	China	NA	plant
CP024510.1	-	CP024504.1	*Klebsiella pneumoniae*	ST323	KL21	NA	NA	NA
CP031262.1	-	CP031257.1	*Klebsiella quasipneumoniae*	ST367	KL1	China	2014	bronchoalveolar lavage fluid

CP035384.1	-	CP035383.1	*Klebsiella pneumoniae*	ST23	KL1	China	2017	blood
FJ876826.1	-	NA	*Klebsiella pneumoniae*	NA	NA	China	NA	NA
CP035776.1	-	CP035777.1	*Klebsiella pneumoniae*	ST37-1LV	KL14	China	2015	rabbit
CP003224.1	-	CP003200.1	*Klebsiella pneumoniae*	ST11	KL103	China	2011	sputum
CP025468.1	-	CP025466.1	*Klebsiella pneumoniae*	ST11	KL103	China	2008	NA
CP021195.1	-	CP021193.1	*Escherichia coli*	-	-	China	2012	NA
CP018999.1	-	CP019000.1	*Escherichia coli*	-	-	China	2013	NA
KU295132.1	-	NA	*Escherichia coli*	NA	NA	USA	NA	rectum swab
KY270849.1	-	NA	*Klebsiella pneumoniae*	NA	NA	China	NA	NA
KX236178.1	-	NA	*Klebsiella pneumoniae*	NA	NA	China	2009	sputum
MG764550.1	-	CP022997.1	*Klebsiella pneumoniae*	ST11	KL47	China	NA	NA
MH477636.1	-	NA	*Klebsiella pneumoniae*	NA	NA	China	2013	NA
MH909340.1	-	GCF_002751155.1	*Klebsiella pneumoniae*	ST11	KL47	China	2013	NA
MH917122.1	-	GCF_003225855.1	*Klebsiella pneumoniae*	ST628	KL2	USA	2015	sputum
MK183753.1	-	CP034076.1	*Klebsiella pneumoniae*	ST15	KL19	China	NA	blood
MK191023.1	-	CP034077.1	*Klebsiella pneumoniae*	ST15	KL19	China	2017	Sputum
LR596806.1	-	LR596806.1	*Klebsiella pneumoniae*	ST11	KL31	UK	NA	NA
17ZR-127	+	RYML00000000	*Klebsiella pneumoniae*	ST11	KL64	ZR	2017	blood
17ZR-133	-	RYKN00000000	*Klebsiella pneumoniae*	ST15	KL19	ZR	2017	blood
17ZR-141	-	RYIT00000000	*Klebsiella pneumoniae*	ST15	KL19	ZR	2017	sputum
17ZR-175	-	RYIQ00000000	*Klebsiella pneumoniae*	ST15	KL19	ZR	2017	blood
17ZR-2	-	RYIM00000000	*Klebsiella pneumoniae*	ST15	KL19	ZR	2017	sputum
17ZR-31	+	RYHQ00000000	*Klebsiella pneumoniae*	ST86	KL2	ZR	2017	sputum
17ZR-84	+	RYHP00000000	*Klebsiella pneumoniae*	ST86	KL2	ZR	2017	sputum
17ZR-91	-	RYGB00000000	*Klebsiella pneumoniae*	ST86	KL2	ZR	2017	sputum

Supplementary Table S2. SNPs of *K. pneumoniae* strains carrying both p17ZR-91-Vir and p17ZR-91-KPC plasmid collected from a hospital in Zhejiang Province, China.

SNP-dists 0.6	17ZR-31	17ZR-84	17ZR-91	17ZR-127	Reference
**17ZR-31**	0	8717	8714	20094	20377
**17ZR-84**	8717	0	23	28712	29094
**17ZR-91**	8714	23	0	28702	29083
**17ZR-127**	20094	28712	28702	0	4118
**Reference**	20377	29094	29083	4118	0

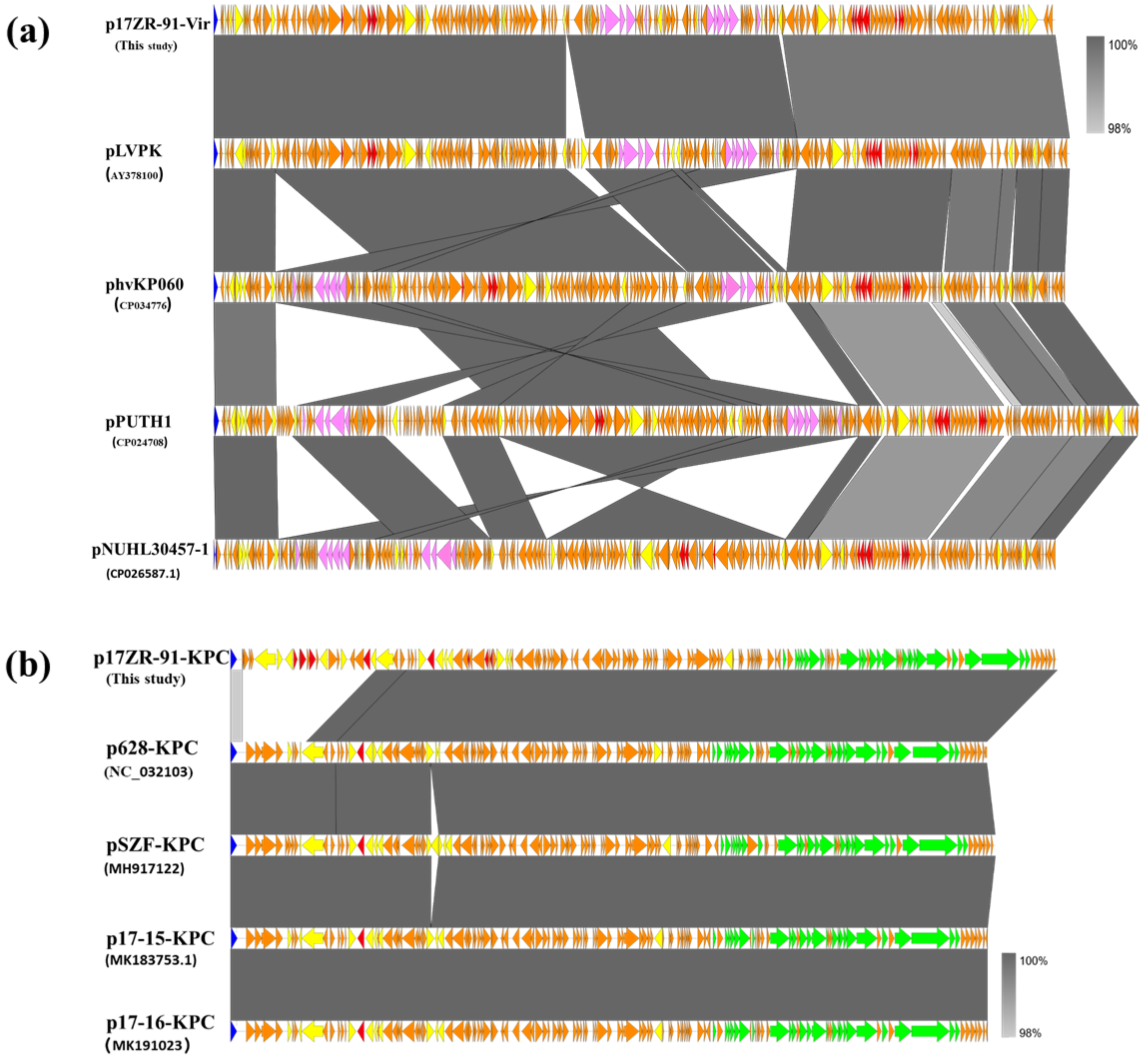

Supplementary Figure S1. Alignment of virulence plasmid p17ZR-91-Vir and *bla*$_{\text{KPC-2}}$-bearing plasmid p17ZR-91-KPC recovered from *K. pneumoniae* 17ZR-91 with similar plasmids in the NCBI database, respectively. (**a**) Alignment of virulence plasmid p17ZR-91-Vir with pLVPK (AY378100), phvKP060 (CP034776), pPUTH1(CP024708) and pNUHL30457-1(CP026587.1) using Easyfig. (**b**) Alignment of *bla*$_{\text{KPC-2}}$-bearing plasmid p17ZR-91-KPC with p628-kpc (NC_032103), pSZF-KPC (MH917122), p17-15-KPC (MK183753) and p17-16-KPC(MK191023) using Easyfig.

```
HR1  AGATCCGAAAAAAGCTAGTTCCGGATCTGCTTCAGCCCCGCCGACGCGCAGCGTCCAAAA  60
HR2  AGATCCGAAAAAAGGTAGTTCCGGATCTGCCGGAACGGCTCCGGCGCGCAGCGGCCCGGC  60
     ************** ***************   * *  * *** ********* **

HR1  AACACAAGATGTAGCGGTGTTCACAGCTGACAGTGACGCTATATGTTGTGTCTCTCCCCT  120
HR2  AACACAAGATGTAGCGGTGTTCCCAGCTGAGGATGACGCTATATGTTGTGTCCCTCCCCT  120
     ********************** *******   ******************* *******

HR1  GCCCTGCGACCGGCCTCTCACCACCCTAAAAGGACGCTTTGCTTATTCCCTGGCCGTGGT  180
HR2  CCCCCGTGACCGGCCGCTCACCACCGGAAAACGCCGCTTTGCTTATTCTCTCACCGTGGT  180
      *** * ******** *********  **** * ************** **  *******

HR1  ACACTTCCGGAAACTTCGCTAAATGTGAAAAGTCGGATCTGCCGGAAACGGTCATCCGAT  240
HR2  ACACTTCCGGAAACTTCGTAAAGCGCGAAAAGTCGGATCCGGACGGAACGGCCGTCCTGT  240
     ******************  **  * ************* *   * ***** * ***  *

HR1  AATTTAATAGCGTGGTTATAATGCTGCTTATTATC     275
HR2  AATTTAACGGCGTGGCTGTATGGCTGCTTGTTATC     275
     *******  ****** * **  ******* *****
```

Supplementary Figure S2. DNA sequence alignment for 275bp homologous regions, HR1 and HR2, located in plasmids p17ZR-91-Vir and p17ZR-91-KPC. Sequence alignment were conducted using multiple sequence alignment tool, Clustal Omega. These two sequences shared 82% (224/275) homology.

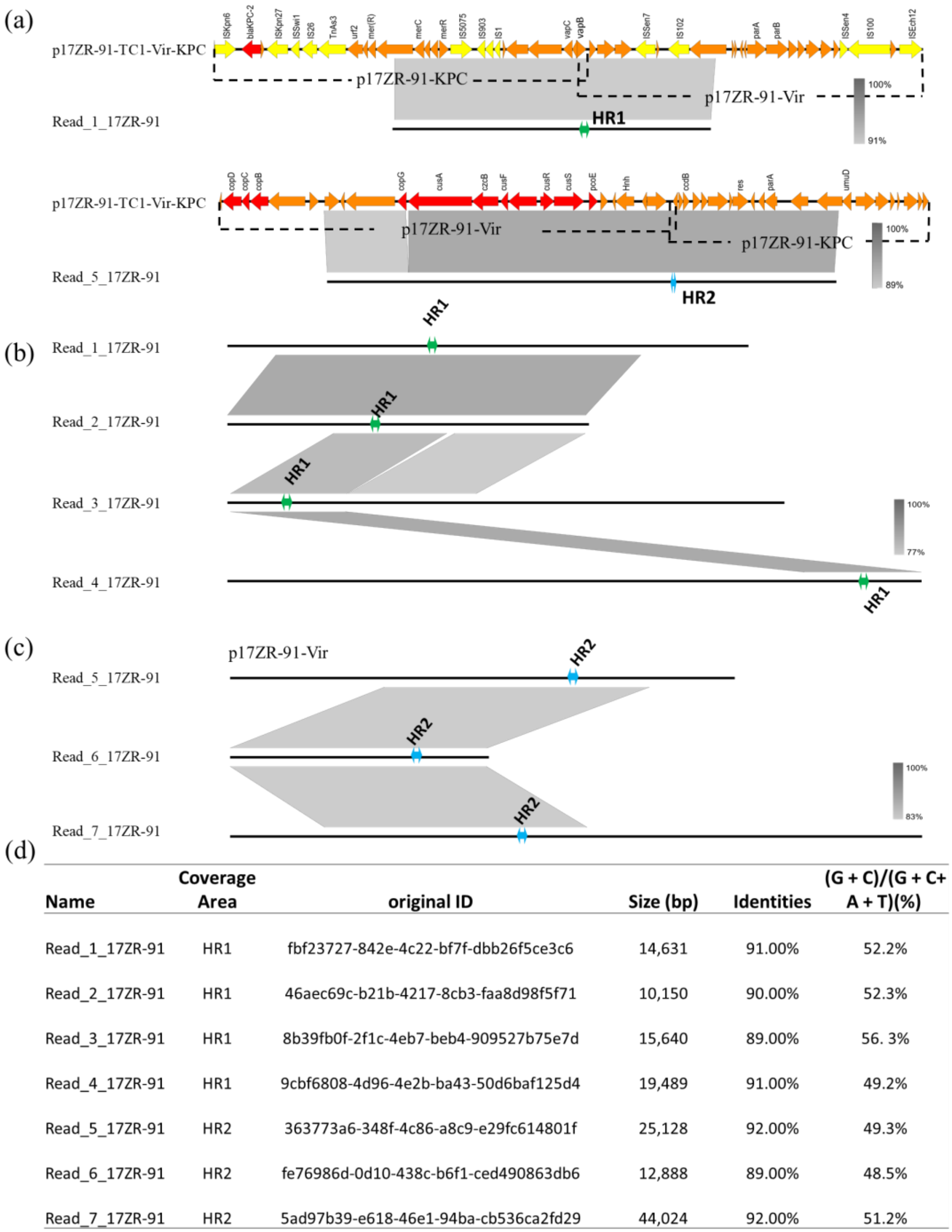

(d)

Name	Coverage Area	original ID	Size (bp)	Identities	(G + C)/(G + C+ A + T)(%)
Read_1_17ZR-91	HR1	fbf23727-842e-4c22-bf7f-dbb26f5ce3c6	14,631	91.00%	52.2%
Read_2_17ZR-91	HR1	46aec69c-b21b-4217-8cb3-faa8d98f5f71	10,150	90.00%	52.3%
Read_3_17ZR-91	HR1	8b39fb0f-2f1c-4eb7-beb4-909527b75e7d	15,640	89.00%	56. 3%
Read_4_17ZR-91	HR1	9cbf6808-4d96-4e2b-ba43-50d6baf125d4	19,489	91.00%	49.2%
Read_5_17ZR-91	HR2	363773a6-348f-4c86-a8c9-e29fc614801f	25,128	92.00%	49.3%
Read_6_17ZR-91	HR2	fe76986d-0d10-438c-b6f1-ced490863db6	12,888	89.00%	48.5%
Read_7_17ZR-91	HR2	5ad97b39-e618-46e1-94ba-cb536ca2fd29	44,024	92.00%	51.2%

Supplementary Figure S3. Nanopore reads covering the fusion region of fusion plasmid p17ZR-91-Vir-KPC. (**a**) Linear alignment between part of p17ZR-91-Vir-KPC and the Nanopore long-read Read_1_17ZR-91 at HR1 region and Read_5_17ZR-91 at HR2 region. Linear alignment among different Nanopore reads that containing HR1 (**b**) or HR2 (**c**) parts of fusion plasmid. (**d**) detail information of 7 reads.

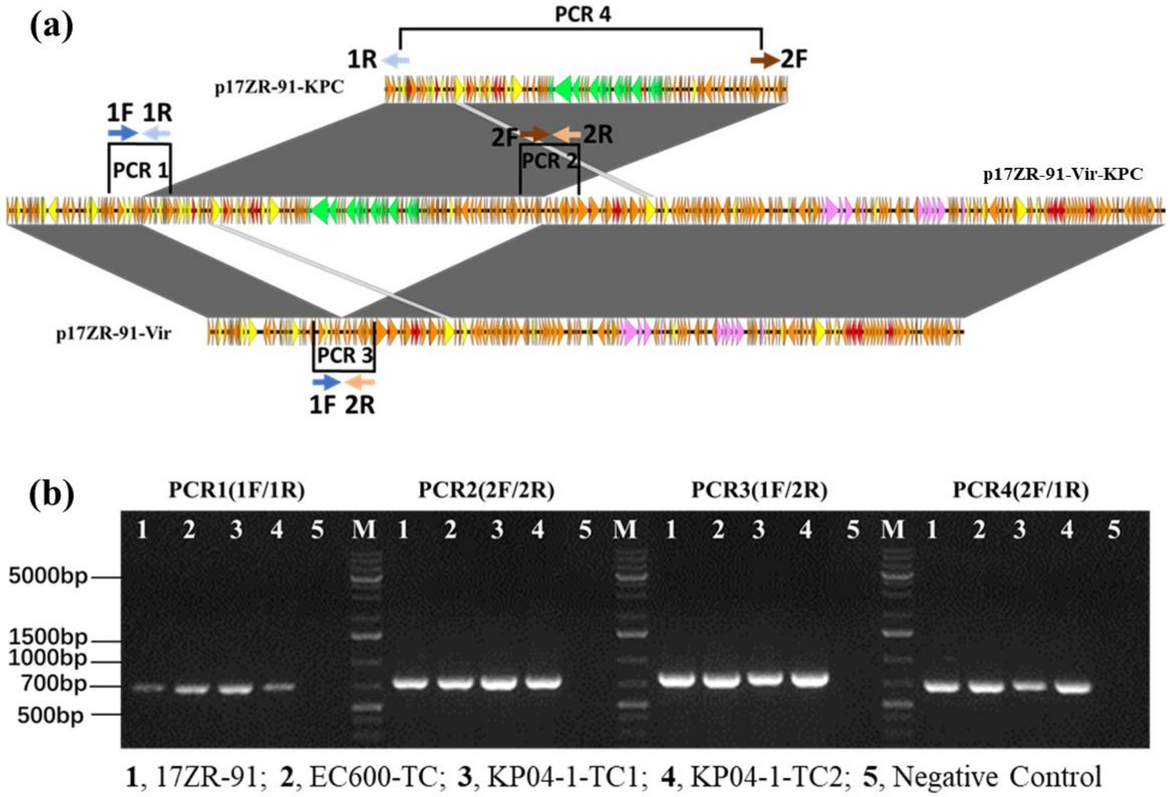

Supplementary Figure S4. PCR assays to confirm the presence of fusion plasmid p17ZR-91-Vir-KPC in different *K. pneumoniae* strains and transconjugants. (**a**) Design of PCR assays to test the presence of fusion plasmid and two non-fusion plasmids in *K. pneumoniae*. PCR1 and PCR2 were designed to confirm the presence of fusion plasmid p17ZR-91-Vir-KPC; PCR3 was designed to confirm the presence of p17ZR-91-Vir; PCR4 was designed to confirm the presence of p17ZR-91-KPC. (**b**) PCR results for different *K. pneumoniae* strains and transconjugants. Being positive in all the four PCR tests indicates the presence of fusion plasmid and two non-fusion plasmids in the same strain.

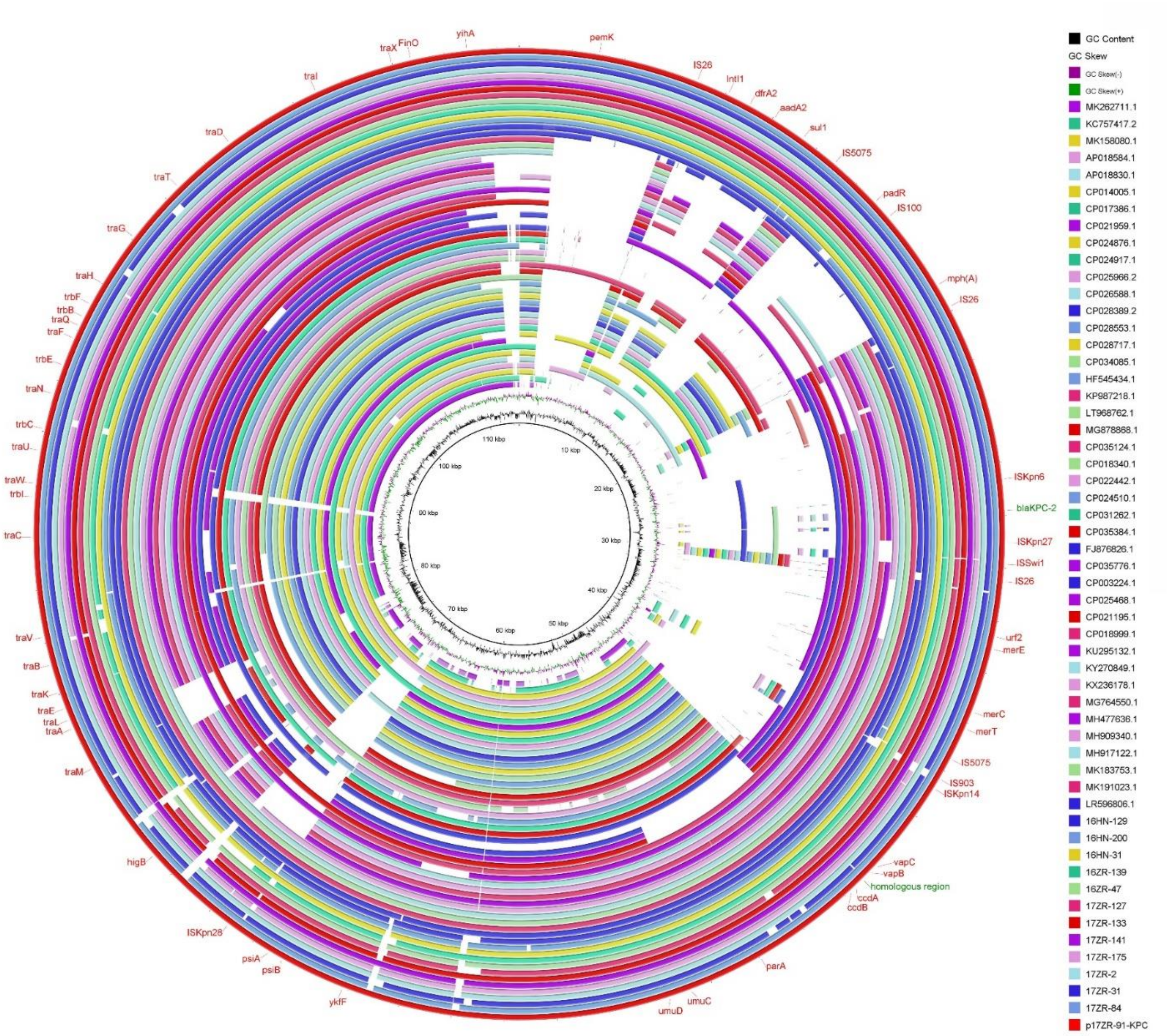

Supplementary Figure S5. Alignment of the *bla*KPC-2-bearing plasmid harbored by 17ZR-91, p17ZR-91-KPC, with other related plasmids recovered from strains in two hospitals in China, and those retrieved from the GenBank. The alignment was performed using BLAST Ring Image Generator (BRIG). Other information of these strains was shown in Supplementary Table S1. Homologous region (HR) was labelled.

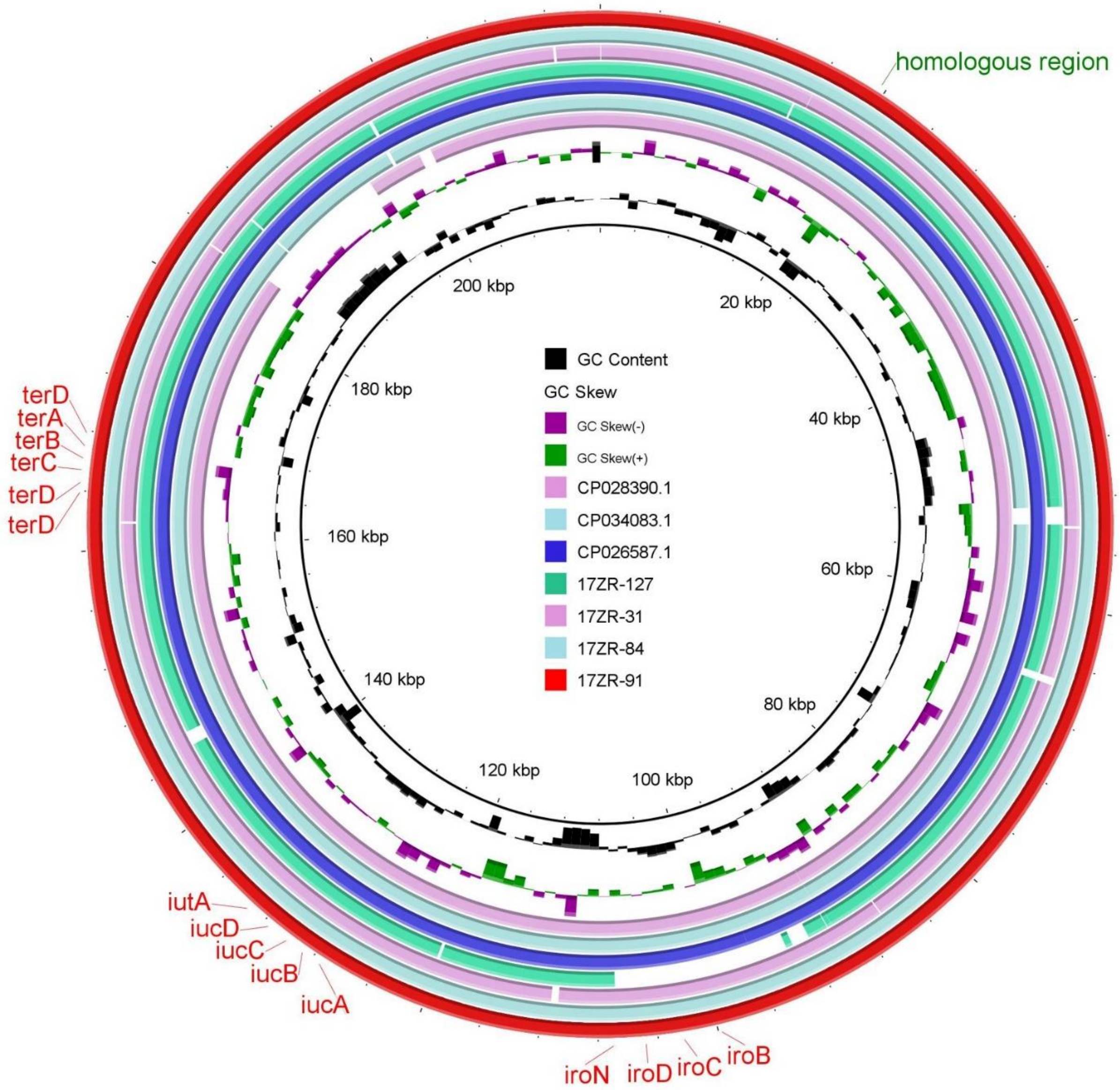

Supplementary Figure S6. Alignment of virulence plasmid from 17ZR-91, p17ZR-91-Vir, with other virulence plasmids from bacterial strains that contained p17ZR-91-KPC, obtained from one hospital in China and the GenBank, respectively. The alignment was performed using BLAST Ring Image Generator (BRIG). Homologous region (HR) was labelled.

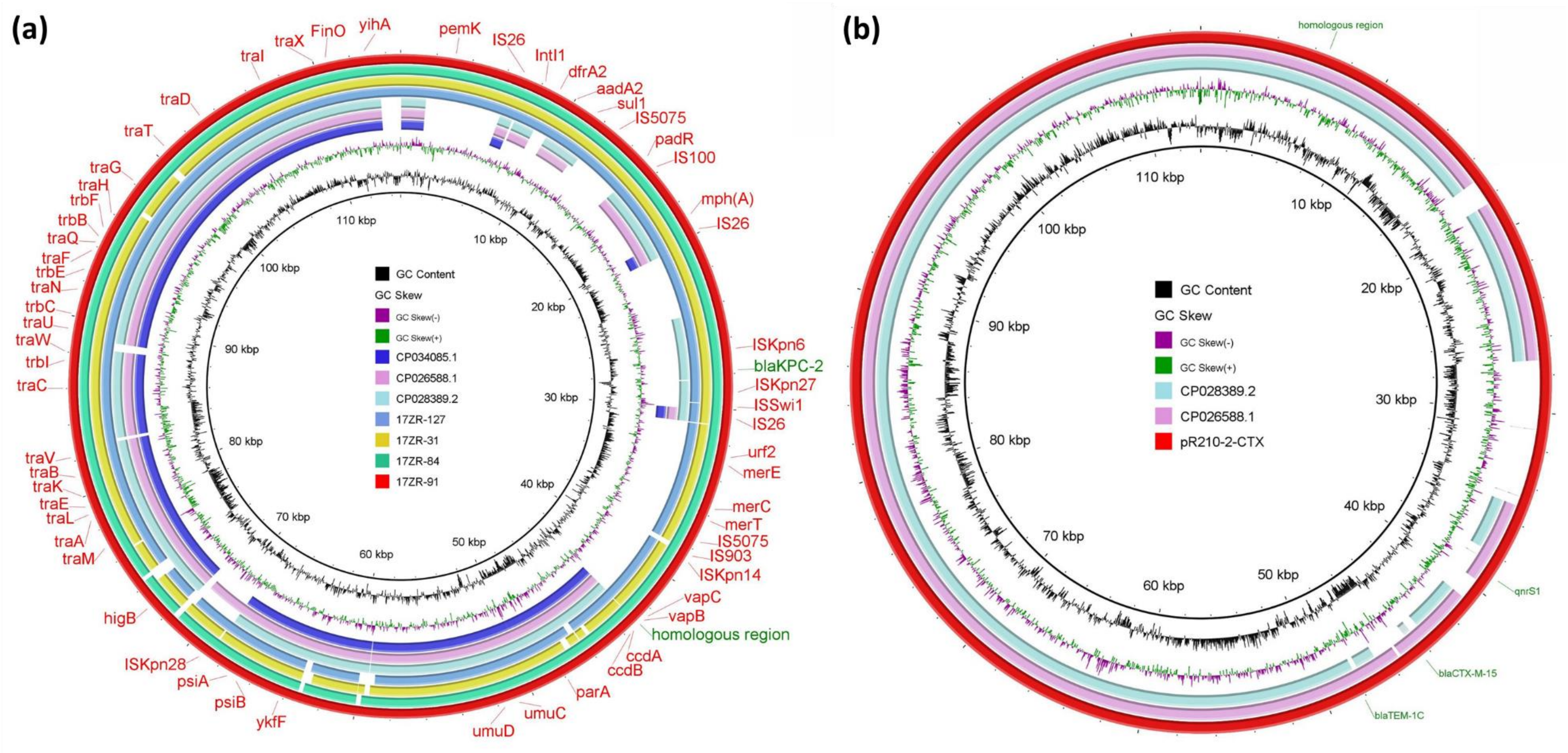

Supplementary Figure S7. Alignment of *bla*$_{\text{KPC-2}}$-bearing plasmid from *K. pneumoniae* strains carrying both p17ZR-91-Vir like and p17ZR-91-KPC like genes. (a) Alignment of *bla*$_{\text{KPC-2}}$-bearing plasmid from six *K. pneumoniae* strains carrying both p17ZR-91-Vir-like and p17ZR-91-KPC-like genes. (b) Alignment of pR210-2-CTX like plasmid with those of the other two *K. pneumoniae* strains documented in the Genbank which also carried the virulence plasmid. The alignment was performed using BLAST Ring Image Generator (BRIG). Four strains from the same hospital exhibited highly similar p17ZR-91-KPC-like plasmids, whereas three strains from the GenBank carried a pR210-2-CTX

(NZ_CP034085.1)-like *bla*$_{\text{CTX-M}}$-bearing plasmid with a backbone similar to that of p17ZR-91-KPC, but contain different MDR regions. Homologous region (HR) was labelled.
